# Altered Spatiotemporal Dynamics of Cortical Activation to Tactile Stimuli in Somatosensory Area 3b and Area 1 of Monkeys after Spinal Cord Injury

**DOI:** 10.1523/ENEURO.0095-16.2016

**Published:** 2016-09-29

**Authors:** Ruiqi Wu, Langting Su, Pai-Feng Yang, Li Min Chen

**Affiliations:** 1 Institute of Imaging Science, Vanderbilt University, Nashville, Tennessee 37232; 2 Department of Radiology and Radiological Sciences, Vanderbilt University Medical Center, Nashville, Tennessee 37232; 3 Department of Psychology, Vanderbilt University, Nashville, Tennessee 37232; 4 Wuhan Institute of Physics and Mathematics, The Chinese Academy of Sciences/State Key Laboratory of Magnetic Resonance and Atomic and Molecular Physics, Wuhan, People’s Republic of China 430071; 5 Psychology Undergraduate Program, Duke University, Durham, North Carolina 27708

**Keywords:** deafferentation, dorsal column lesion, hand, new world monkeys, touch

## Abstract

Reactivation of deafferented cortex plays a key role in mediating the recovery of lost functions, although the precise mechanism is not fully understood. This study simultaneously characterized the dynamic spatiotemporal features of tactile responses in areas 3b and 1 before and 6–8 weeks after partial dorsal column lesion (DCL), and examined how the reactivation relates to the recovery of simple hand use in squirrel monkeys. A combination of high spatiotemporal resolution functional intrinsic optical imaging, microelectrode mapping, behavioral assessment, and tracer histology methods were used. Compared with the normal cortex, we found that the responses of deafferented areas 3b and 1 to 3 s of continuous 8 Hz tactile stimulation of a single digit were significantly weaker and more transient. This finding indicates a loss of response to sustained tactile stimuli. The activation area enlarged for areas 3b and 1 in both directions along digit representation (medial–lateral) and across areas (anterior–posterior). All subjects showed behavioral deficits in a food reaching-grasping-retrieving task within the first 5 weeks after DCL, but recovered at the time when optical images were acquired. Summarily, we showed that these populations of cortical neurons responded to peripheral tactile inputs, albeit in significantly altered manners in each area, several weeks after deafferentation. We propose that compromised ascending driven inputs, impaired lateral inhibition, and local integration of input signals may account for the altered spatiotemporal dynamics of the reactivated areas 3b and 1 cortices. Further investigation with large sample sizes is needed to fully characterize the effects of deafferentation on area 1 activation size.

## Significance Statement

This study simultaneously characterized the spatiotemporal dynamics of cortical responses to peripheral tactile stimuli in deafferented area 3b and area 1 following partial dorsal column lesion in temporal and spatial resolutions that have not been achieved before. Deafferented areas 3b and 1 lost their ability to respond to sustained tactile inputs and recruited surrounding cortex for processing these inputs, but in different manners spatially and temporally. This study provides novel insights into how the modules of individual digit representations in area 3b and area 1 adapt to or compensate for a loss of sensory inputs and work together to process peripheral tactile inputs to contribute to behavioral recovery of impaired hand uses in nonhuman primates following spinal cord injury.

## Introduction

Humans and other adult mammals typically demonstrate various degrees of functional and behavioral recovery over a period of days to months after partial or almost complete damage to the CNS (Gilman and Denny-Brown, 1966; Goldberger and Murray, 1978; Pubols and Goldberger, 1980; Finger and Almli, 1985; Jain et al., 1997; Kaas, 2000). Specifically, after spinal cord injury (SCI), input-deprived brain regions, such as the primary somatosensory cortex (SI), regain their responsiveness to stimuli (reactivation) over time but with an abnormal somatotopy (i.e., reorganization; Jain et al., 1997, 1998; Kaas, 2000; Kaas and Collins, 2003). These plastic changes are believed to play a key role in mediating functional and behavioral recovery after injury, even though the underlying mechanisms are not fully understood. Following SCI, an effective way of exploring the mechanisms of plasticity and their contributions to behavioral recovery is to monitor parallel changes in the brain and behavior over time, to characterize how reactivated cortex processes peripheral inputs, and ultimately to determine the relationships between functions of reactivated cortex and the corresponding behavioral improvement of individual subjects. Execution of normal sensorimotor behaviors in primates, such as hand use, engages distributed cortical and subcortical regions in a functionally specific manner. Thus, it is reasonable to predict that the recovery of impaired hand use after SCI is likely to be mediated by the collective efforts of different regions along the information processing and modulatory pathways. In this context, it is essential to simultaneously measure the spatiotemporal dynamics of multiple areas in action. To date, however, few studies have directly examined how different somatosensory cortical areas work together to restore lost cortical functions or to compensate for dysfunction after SCI (Merzenich et al., 1983; Tandon et al., 2009; Bowes et al., 2013). Previous mapping studies, conducted simultaneously with high-resolution fMRI and optical imaging of intrinsic signals, provided the first line of evidence supporting the behavioral relevance of the extent of somatotopic reorganization of digit (D) representation in areas 3b, 1, and S2 after a partial dorsal column lesion (DCL; Yang et al., 2014), and the degree of afferent disruption (Qi et al., 2011; Chen et al., 2012). What remain unknown are the fine-grained spatiotemporal features of the activity and/or the responsiveness to incoming tactile inputs in those reactivated and reorganized cortical regions after disruptions of ascending spinal afferents. Furthermore, little is known about how functionally related multiple deafferented cortical areas work together to process tactile inputs and contribute to the recovery of impaired hand use after SCI.

To address these questions, in the present study we characterized the spatiotemporal dynamics of the response in partially deafferented and reactivated area 3b and area 1 to tactile stimulation, and explored the relationship between response property changes and hand use in squirrel monkeys by comparing functional optical imaging of intrinsic signals (IOSs) before and 6–8 weeks after DCL. The partial DCL of high cervical spinal cord was used in the present study because it disrupts a somatic sensory pathway (dorsal column) while leaving the motor pathway intact, introduces reactivation and reorganization of sensory cortices, and causes hand use impairments (Jain et al., 1997; Darian-Smith and Ciferri, 2006; Kaas et al., 2008; Qi et al., 2013). IOS is a powerful functional imaging technique that detects similar hemodynamic-related signals as BOLD signal, but with superior spatial (∼100 μm) and temporal (∼250 ms) resolution. Specifically, by simultaneously characterizing the spatiotemporal features of IOSs to repeated tactile stimuli in normal versus deafferented cortex following SCI in anesthetized monkeys, we specifically asked the following questions: (1) how do digit modules in partially deafferented areas 3b and 1 process tactile inputs as individual areas and as a group in space and time after SCI?; (2) do deafferented and reactivated areas 3b and 1 behave in similar manners?; and (3) how do these changes in response properties relate to hand use behavior?

## Materials and Methods

### Animal preparation

Six adult male squirrel monkeys (*Saimiri sciureus*) were included in this study, three of which underwent partial dorsal column section (i.e., DCL). In two monkeys (SM-CHI and SM-CHA), somatosensory areas 3b and 1 of one hemisphere were studied before and 6 weeks after unilateral SCI (DCL) at the C4–C5 level. In one monkey (SM-COA), both hemispheres were studied at 8 weeks after DCL with the side ipsilateral to the lesion used as normal cortex. Three naive monkeys (SM-PUA, SM-GUA, and SM-POT) were studied as normal controls. For optical imaging (OI) and electrophysiological mapping experiments, animals were initially sedated with ketamine hydrochloride (10 mg/kg) and atropine sulfate (0.05 mg/kg, i.m.), and then anesthetized with isoflurane (0.7–1.2%). During functional imaging, animals were maintained at a light (0.7–0.8%) and stable level of anesthesia. Animals were artificially ventilated to maintain an end-tidal CO_2_ level of 4%. Rectal temperature was maintained between 37.5°C and 38.5°C by means of a circulating water blanket. Heart rate, respiration, pulse oximetry, end-tidal CO_2_, and EKG were continuously monitored during the entire procedure. Under aseptic conditions and after the level of anesthesia reached a surgical plane, a 12-mm-diameter craniotomy was made over the central sulcus where the SI resides, and dura was removed to expose cortex for OI, and microelectrode mapping and recording. Digital pictures of the brain surface were taken, and the surface blood vessel patterns were used for coregistration purposes. All procedures were conducted in accordance with National Institutes of Health guidelines and were approved by the Institutional Animal Care and Use Committees of Vanderbilt University.

### Stimulation protocol

The hand and fingers were stabilized with modeling clay, palm side up, leaving glabrous skin of the distal finger pads available for stimulation. Pegs imbedded in the clay were glued to fingernails to hold the fingers in place. A probe 2 mm in diameter attached to a piezoceramic bender (Noliac), driven by an S88 Grass stimulator, delivered 8 Hz (with 30 ms pulse duration) vibrotactile stimuli (vertical trapezoid indentations with 0.43 mm displacement) to each distal finger pad. Each stimulation epoch lasted 3.5 s.

### OI data acquisition and analyses

Functional optical images were collected using an Imager 3001 system (Optical Imaging) with 630 nm illumination. A blood vessel map was collected with 570 nm illumination. In a typical imaging run, three digits (e.g., D1, D2, and D3) were stimulated separately in a randomly interleaved manner. Fifty trials were usually collected per stimulus condition, and 15 image frames (250 ms per frame) were acquired during a 3.75 s imaging trial. Intrinsic optical image data acquisition started 250 ms prior to stimulus onset. The interstimulus interval was 7 s. Detailed surgical and OI procedures were similar to those previously reported (Chen et al., 2003, 2005,2007).

Raw optical images were preprocessed in MATLAB (MathWorks; RRID: SCR_001622) with custom-written scripts. The luminous intensity of each image frame was corrected to reduce irregularity resulting from brain pulsation during data acquisition. We first calculated the raw activity maps by subtracting the first prestimulus frame from each subsequent frame of each stimulus condition, and then averaged all 14 activity maps across all trials to get a rough mean activity map. Background (nonactivated regions) images were lined out based on the raw activity maps. We calculated the average background intensities of each frame (*I*_each_baseline_) and each trial (*I*_trial_baseline_). Then, the intensity of each pixel in the raw image within each trial was subtracted by the mean background intensity of that particular image, and then the mean background intensity of all 15 image frames was added (*I*_corrected_ = *I*_raw_ − *I*_each_baseline_ + *I*_trail_baseline_). All subsequent analyses were performed on the intensity-corrected images. Because absolute raw OI signal reflectance (*R*) values varied significantly within and across imaging sessions, depending on lighting conditions and specifics of the preparation, we used the percentage of signal change in our quantifications. A spatial mean filter [window size = 5 × 5 pixels (70 × 70 μm^2^)] was applied. Activity maps were obtained by subtracting the first prestimulus frame from each subsequent frame of each stimulus condition (*dR*/*R* = (*R*_stimulus_frame_ (*ti*) − *R*_first_frame_)/*R*_first_frame_, where *dR* is the signal change and (*ti*) is time stamp). Since cortical activation of tactile stimuli is reflected by OI signal decreases (i.e., increased OI signal absorbance; Chen et al., 2005), we reversed the plot *y*-axis as −dR/R (see Figures 2–7 to better visualize the signal increases). To increase imaging signal-to-noise ratio, we averaged all 14 activity maps (or frames) across all trials to generate one series of activation maps showing the temporal development of the cortical response to stimuli (for one example, see Fig. 2*B*). To generate a summary activation map for each stimulus condition, we averaged together the five temporally contiguous image frames showing peak activation (for one example, see Fig. 2*Ab*). Activation maps were generated for each digit for each imaging session (prelesion or postlesion). In functional maps, dark pixels represent a response greater than that of the prestimulus image. All of the presented OI maps in the same figure were displayed with the same linear intensity scale for comparison purposes.

### Measurement of activation size

The size of single-digit activation was quantified, based on the averaged activation maps, for each digit in areas 3b and 1 in prelesion (normal) and postlesion (lesioned) conditions. The selection of the regions of interest (ROIs) for each digit in area 3b or area 1 was based on the OI activation maps and the digit representation map later determined electrophysiologically. We first manually defined ROIs in area 3b and area 1 that were a bit larger than the dark activated region in the OI map. Next, we considered pixels that exhibited greater than −0.012% signal change within each ROI as activated pixels. The total number of activated pixels (*dR*/*R* less than −0.12‰) in the ROI was then converted into square millimeters based on the pixel resolution (0.0139 × 0.0139 mm^2^; matrix size = 504 × 504; FOV = 7 × 7 mm^2^) for further quantification. Only those digits with activation detected in both prelesion and postlesion conditions were included in the quantification of activation size changes after DCL (see Fig. 5).

### Quantification of time courses of optical signals

Time courses of OI signals were extracted from the center of each activated region and a nonactivated control region (ROI size, 20 × 20 pixels) for quantifying amplitudes (e.g., peak) and temporal profiles (e.g., time to peak) of responses to stimuli, and then were averaged across trials for each stimulation condition. A control region was defined as a nearby cortical area (in either area 3b or area 1) showing no activation to stimuli. Time courses extracted from this region served as built-in signal fluctuation controls for activated regions.

### Statistical analysis

Group statistical analysis was performed on the following eight measures: activation magnitude, activation size, time to amplitude peak, time to peak activation size, amplitude and area changes between 2.0 and 3.5 s after stimulus onset, the full-width at half-maximum (FWHM) of the lateral–medial and anterior–posterior axis of single-digit activation in area 3b and area 1 in normal and postlesion deafferented conditions. Time courses of area size development were normalized by using the peak activation size as a 100% reference before statistical calculation. The time-to-peak measure is determined by averaging all peak time points of each individual run across all subjects. Unpaired *t* tests were performed on activation amplitudes, time to peak amplitude, time to peak activation size, amplitude changes between 2.0 and 3.5 s after stimulus onset, and area changes between 2.0 and 3.5 s after stimulus onset. For area 3b, 25 digit cases before DCL (df = 24) and 7 digit cases after DCL (df = 6) were summarized; for area 1, 19 digit cases before DCL (df = 18) and 6 digit cases after DCL (df = 5) were summarized. Paired *t* tests were performed on the comparison of peak activation sizes and FWHM measures in normal versus lesioned conditions. There were six digit pairs in area 3b, and three digit pairs in area 1. The FWHM of single-digit activation focus in both medial-to-lateral (along the digit representation) and anterior-to-posterior (across cortical area) directions were quantified by extracting signal amplitude along the medial-to-lateral and anterior-to-posterior lines cross at the center of activation. A *p* value of <0.05 was considered statistically significant.

To maximize the sensitivity in detecting condition differences (lesion vs normal) in OI amplitudes at the group level, we pooled measures of all digits (D1–D5) from all animals (see Fig. 6). The actual digit contributions from individual animals are summarized in Table 1. The measures of activation area, however, varied drastically across digits (from ∼2 to ∼6 mm in normal conditions; see Fig. 5C). Thus, only the digits whose activations were detected in both prelesion and postlesion conditions were included in the group quantification and comparisons of changes in activation areas (see Figs. 6, 7).

**Table 1:** —Summary of data source of normal animals (prelesion, SM-CHI and SM-CHA; normal control, SM-PUA, SM-GUA, and SM-POT; ipsilateral, SM-COA) and lesioned animals (postlesion, SM-CHI, SM-CHA, and SM-COA) included in this study

	SM-CHI	SM-CHA	SM-PAU	SM-GUA	SM-POT	SM-COA	Total digits
Normal area 1	D3, D5	D1, D2, D4, D5	D1, D2, D4	D1, D2, D3, D4, D5	D2, D3, D4, D5	D2	19
Lesioned area 1	D2, D4	D1, D2, D3, D4	Null	Null	Null	Null	6
Normal area 3b	D1, D2, D3	D1, D2, D3, D4, D5	D1, D2, D3, D4	D1, D2, D3, D4, D5	D1, D2, D3, D4, D5	D1, D2, D3	25
Lesioned area 3b	D2, D4	D1, D2	Null	Null	Null	D1, D2, D3	7

### Dorsal column lesion

Unilateral DCL of the cervical spinal cord was performed under surgical-level isoflurane (1–2%) anesthesia. Under sterile conditions, a dorsal portion of the cervical spinal cord was exposed, and the dorsal columns were sectioned on one side with a pair of fine surgical scissors at C4–C5 segment level. The dura was replaced with Gelfilm and covered with Gelfoam. The surgical opening was enclosed with several layers of sutures. Additional details about surgical and postsurgical procedures can be found in previous publications (Qi et al., 2011; Chen et al., 2012; Yang et al., 2014). The level and extent of the lesions were determined according to the cervical vertebra, and later were confirmed with track-tracing histology.

### Histological evaluation of the lesion level and disruption of dorsal column afferents

To determine at which cervical level and to what degree the dorsal column afferents were disrupted, we injected cholera toxin B subunit (CTB) tracer (5 μm/site) into the finger pads of D1, D3, and D5 of both hands. By using the CTB terminals on the normal side of the spinal cord as a segmental reference, we then determined at which digit level the lesion was placed. Similarly, by comparing the sizes of CTB-stained regions in the cuneate nuclei of the brainstem and treating the normal unlesioned side as 100%, we quantified the percentage of afferent disruption for each animal (see Fig. 7). For details on the quantification method and example, see the study by Qi et al. (2011). Using this method, we determined that the percentage of digit afferent disruption was 63% for SM-CHA, 68% for SM-CHI, and 98% for SM-COA.

### Behavior observation, training, and testing

The hand use behavior of the animal was evaluated from the following two aspects: home-cage activity and food reaching-grasping-retrieving behavior. After unilateral DCL, all animals showed hand use impairments, which are characterized by an unwillingness to use an injured hand for cage climbing, abnormal hand posture on holding food, and more false-positive results of food-retrieving trials. These abnormal hand uses were not indications of motor impairments, because the monkeys still used their affected hand normally in cage-climbing and food-retrieving activities when they were forced (e.g., when the good unaffected hand was occupied with treats). All three animals included in this study were also trained in their home cage on food reaching-grasping-retrieving tasks prior to DCL. The task involved reaching and retrieving a small sugar pellet from a well on a modified Kluver board (15.2 × 9.9 cm). Four wells (well 1 to well 4) with different depths (0.14, 0.28, 0.38, and 0.64 cm, respectively) and diameters (0.78, 1.20, 1.14, and 1.14 cm, respectively) were created to introduce incremental difficulties into the task, with well 1 being the easiest and well 4 being the hardest. Within each behavioral session, the performances of an animal on 80 task trials (20 trials for each well) were recorded using a high-speed (60 frames/s) video camera. Behavioral data were collected three times before the lesion (one mean value generated) and then daily after the lesion (after skipping the first 3 d immediately after lesion). Daily sessions were summed to generate a single performance score for each week. There were circumstances, such as the recovery period from an anesthesia event, where the behavioral data collection may have been disrupted. The procedures were similar to those detailed in previous reports (Qi et al., 2013, 2014). A successful food retrieval rate and the number of digit flexes were measured for quantitative scoring. A successful trial is one in which the sugar pellet was grasped and transferred into the mouth using the designated hand. The success rate for each well was calculated by dividing the number of pellets retrieved by the total number of trails. On the other hand, a single flexion of the digits for a successfully retrieved pellet was considered to be a perfect score. The number of digit flexes per successful trial for each well was calculated using the following formula: digit flexes/successful trial = total number of digit flexes in successful trials/number of successful trails.

## Results

### Dynamic spatiotemporal changes of cortical response to stimulation in area 3b and area 1 after DCL

**Figure 1.**
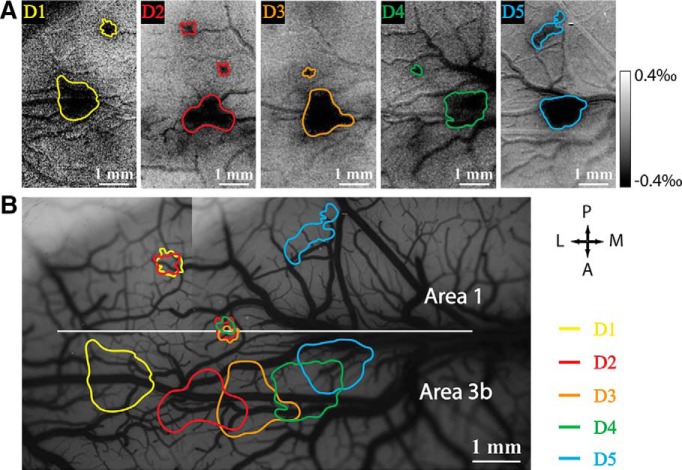
Individual digit representation in somatosensory area 3b and area 1 revealed by optical imaging intrinsic signals in one subject, SM-CHA. ***A***, Optical imaging activations (dark spots) of D1–D5 in response to 8 Hz vibrotactile stimulation. ***B***, Composed activation map shows the somatotopy of the individual digit activations (color outlines) on the blood vessel map.

**Figure 2.**
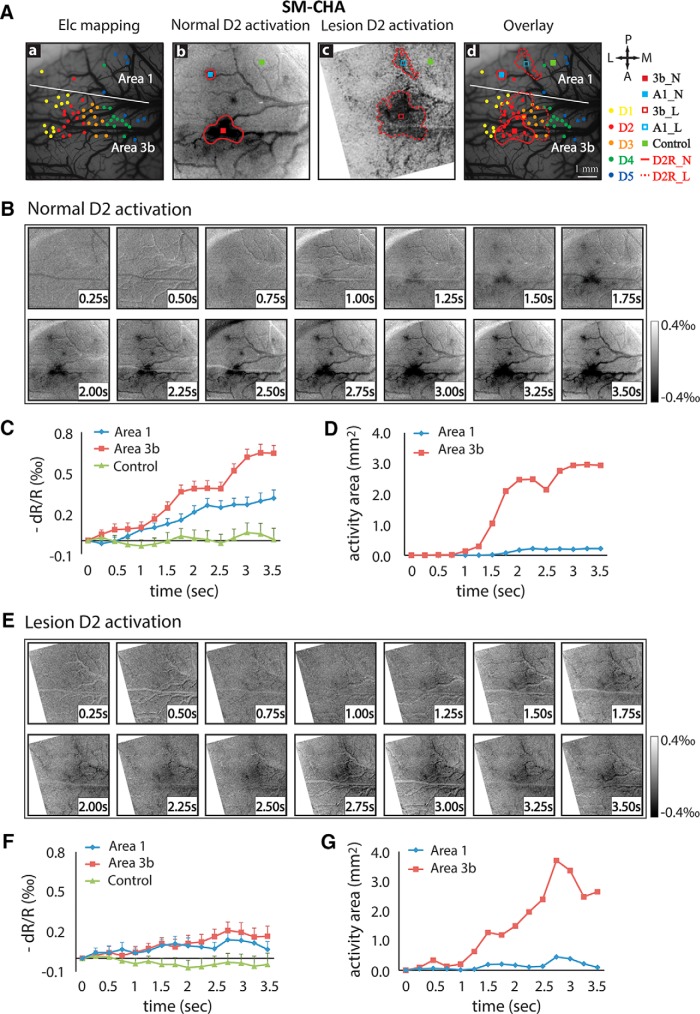
Spatiotemporal dynamics of optical imaging activation in response to single-digit stimulation in somatosensory areas 3b and 1 before and after a DCL in one representative monkey (SM-CHA). ***Aa–Ad***, Postlesion electrophysiological map of digit representation (***Aa***), D2 mean activation map before DCL (***Ab***), D2 mean activation map after DCL (***Ac***), and overlay of ***Aa***, ***Ab***, and ***Ac*** (***Ad***). Electrode penetration sites and properties of the receptive fields are indicated by color dots, and the digit activations are circled by color outlines. Receptive field properties: yellow dots, D1; magenta dots, D2; orange dots, D3; green dots, D4; and blue dots, D5. Full red outlines (D2R_N) and dash red outlines (D2R_L) indicate D2 activation before and after DCL, respectively. Squares show regions for extracting optical signals (see squares in ***Ab***, ***Ac***, and ***Ad***): center of activated area 3b before DCL, red full squares (3b_N); center of activated area 1 before DCL, blue full squares (A1_N); center of activated area 3b after DCL, red dash squares (3b_L); center of activated area 1 after DCL, blue dash squares (A1_L); and one nonactivated control region, green squares. ***B***, First frame-subtracted serial optical images show the development of D2 activations over a 3.5 s vibrotactile stimulation period in areas 3b and 1 before DCL. ***C***, Prelesion time course development of optical signals extracted from area 1 (blue line), area 3b (red line), and a control location (green line). ***D***, Prelesion development of activation sizes in area 1 (blue line) and area 3b (red line) during stimulation. ***E***, First frame-subtracted serial optical images show the development of D2 activations over a 3.5 s stimulation period after DCL. ***F***, Postlesion time course development of optical signals in area 3b (red line), area 1 (blue line), and control (green line). ***G***, Postlesion temporal profile of activation sizes in area 3b (red line) and area 1 (blue line). Error bars indicate the SE.

Before the lesion, we obtained activation maps of individual digits to vibrotactile stimulation. Consistent with our previous observations, single-digit activations organized in an orderly somatotopic manner in area 3b and area 1 (Fig. 1*A*, D1–D5). Presentation of the series of images acquired at a rate of 250 ms/frame showed the temporal development of optical activation maps to D2 stimulation in both area 3b and area 1 over a period of 3.5 s (Fig. 2*B*). Responses in area 3b (bottom half of the field of view) and area 1 (top half of the field of view) started as weak and diffuse at 0.75 s after stimulus onset, and then became focal and strong by 1.75 s after stimulus onset and remained so until the end of stimulation (Fig. 2*B*). The time courses of the OI signals extracted from the activation foci in area 3b and area 1 exhibited similar temporal features (Fig. 2*C*). The signals started to increase ∼0.75 s after stimulus onset and reached a peak at ∼3.25 s (Fig. 2*C*, red and blue curves). The peak signal amplitude of area 3b (0.7‰) was about two times stronger than that of area 1 (0.3‰). The signals of one nonactivated area 1 control region fluctuated along the zero line (Fig. 2*C*, green curve). Activation size increased drastically 1.5 s after stimulus onset, but the size of area 3b activation (3.0 mm^2^) was 10 times bigger than that of area 1 (0.3 mm^2^) at the end of stimulation (Fig. 2*D*, compare red curves, blue curves).

After a unilateral section of left dorsal column at the C4–C5 level, which disrupted 63% of the afferents from the digits, the response to identical stimuli weakened, the center of activation shifted (Fig. 2*Ab*, *Ac*), and the size of activation increased, indicating a more diffused activation after lesion (Fig. 2*E*). For example, the darkness of the D2 activation in area 3b after lesion (Fig. 2*Ac*, red dash outline) was much lighter and less focal compared with the prelesion D2 activation (Fig. 2*Ab*, red dash outline). The activation center shifted toward the border palm region between area 3b and area 1. Response territories also expanded to the neighboring D3 region (Fig. 2*Ad*, orange dots enclosed in the red dash outline). Presentation of the series of image frames clearly illustrates the different spatiotemporal dynamics of area 3b and area 1 response to identical D2 stimulation (Fig. 2*E*). Responses started in areas 3b and area 1 at 1.25 s after stimulus onset (Fig. 2*F*, red and blue curves). The amplitude OI signal changes (peak at ∼0.2‰) were smaller than responses in the prelesion condition (with a peak at ∼0.7‰ for area 3b, and at 0.3‰ for area 1), but that peak occurred earlier (2.75 s) than normal (3.25 s for area 3b and 3.5 s for area 1). The activation area of both area 3b and area 1 started to increase at 1.25 s after stimulus onset and peaked earlier than normal (2.75 vs 3.25 s; Fig. 2*D*,*G*, compare red curves, blue curves). The activation size for area 3b appeared to be bigger than normal, as well.

### Similar spatiotemporal dynamic changes after DCL were observed in other two monkeys

**Figure 3.**
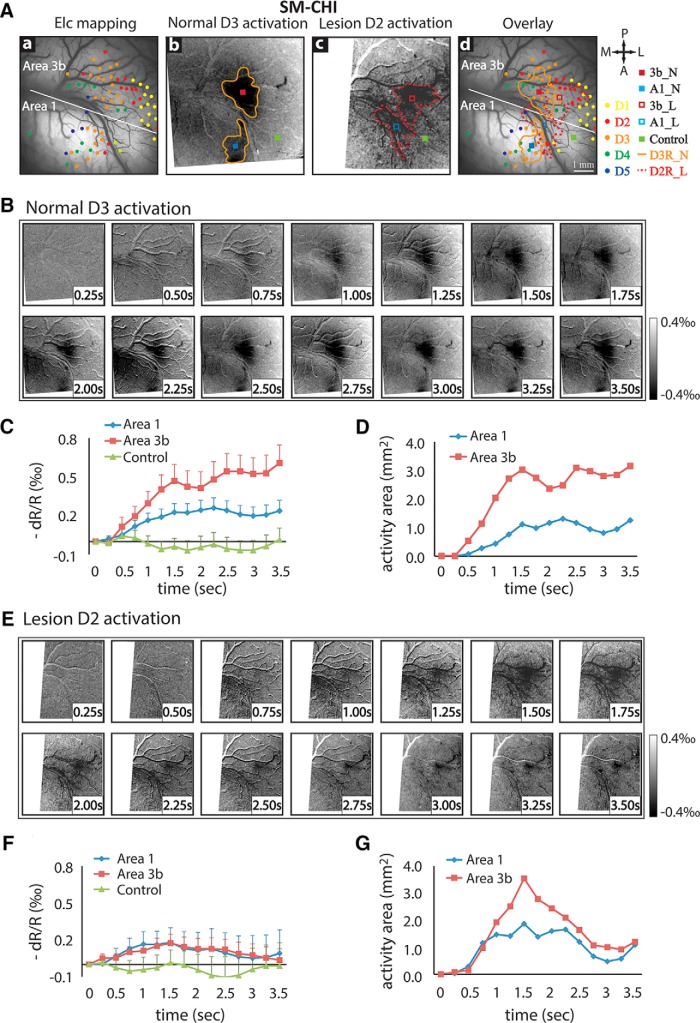
Spatiotemporal dynamics of optical imaging activation to single-digit stimulation in somatosensory areas 3b and 1 in prelesion and postlesion conditions in the second monkey (SM-CHI). Same format as Figure 2, except data are from vibrotactile stimulation of D3 prelesion and D2 6 weeks postlesion.

**Figure 4.**
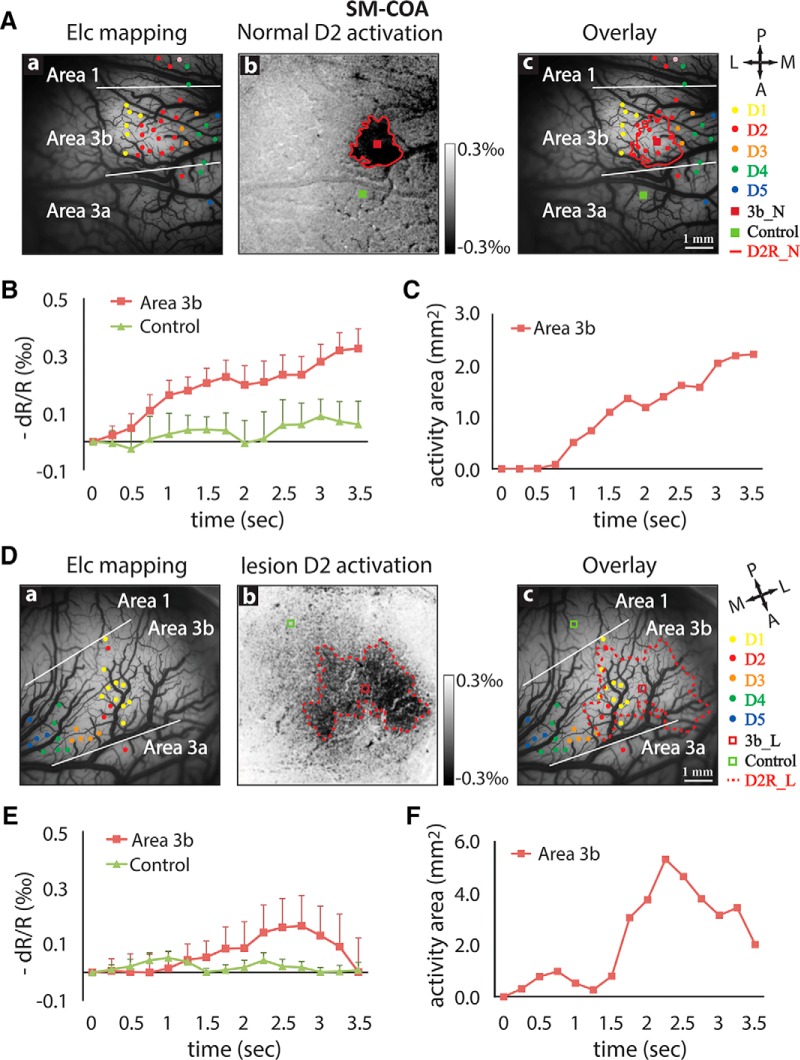
Spatiotemporal dynamics of optical imaging activation to single-digit (D2) stimulation in normal and lesioned somatosensory area 3b in a third monkey (SM-COA). ***A***, Normal cortex. ***Aa–Ac***, Electrophysiological map of digit representation (***Aa***), D2 activation map (blue outline) of the vibrotactile stimuli in contralateral normal cortex (***Ab***), and the overlay (***Ac***). ***B***, Time courses of the optical imaging signals of D2 activation in area 3b (blue outline) and one control location (red outline). ***C***, Temporal profile of the size changes of D2 area 3b activation. ***D***, Deafferented cortex. Electrophysiology digit representation map (***Da***), average D2 activation map (***Db***), and overlap map (***Dc***) at 8 weeks postlesion. ***E***, Time courses of the optical signals of D2 activation in area 3b (blue outline) and in one control region (red outline). ***F***, Change of D2 activation size in area 3b during stimulation ipsilateral to the side of the spinal cord injury. Error bars indicate the SE.

We observed similar spatiotemporal dynamic changes in area 3b and area 1 in response to vibrotactile stimulation after DCLs in two other monkeys (SM-CHI and SM-COA; Figs. 3, 4). For subject SM-CHI, prelesion D3 stimulus-evoked activations in areas 3b and 1 were focal (Fig. 3*Ab*) and located at appropriate digit representation regions, as confirmed by the electrophysiological map (Fig. 3*Ad*, compare activation outline, color dots). Temporal development of optical signal amplitudes and activation size changes in area 3b were comparable to those observed in the first subject, SM-CHA (compare Fig. 3*C*,*D*, 2*C*,*D* ). After lesion, we again observed weak, early peaking, and short (more transient) responses in both area 3b and area 1 (Fig. 3*E–G*). For example, the optical signals reached peak at ∼1.5 s and then declined until the end of stimulus presentation. Signal amplitude decreases, however, were more drastic for area 3b (∼0.6‰ to ∼0.2‰) than area 1 (∼0.3‰ to ∼0.2‰). Activation size showed similar temporal profiles for both area 3b and area 1 (Fig. 3*G*). For the third monkey SM-COA, we also observed similar spatiotemporal dynamic changes in area 3b after a lesion that disrupted 98% of dorsal column afferents (Fig. 4*D–F*). In this subject, area 1 activation was not detected. As a control at 8 weeks postlesion, we also studied the intact contralateral pathway where D2 stimulus evoked focal activation in area 3b (Fig. 4*A–C*).

### Group quantification of the spatiotemporal feature changes of responses after DCL

**Figure 5.**
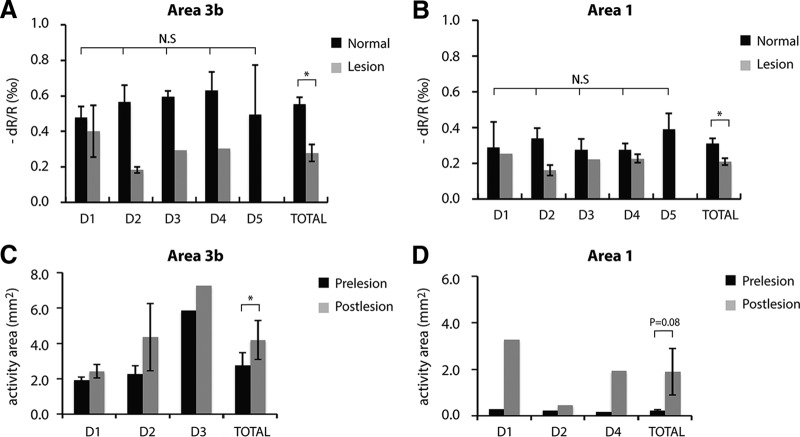
***A–D***, Plots of individual digit and group averaged OIS signal amplitudes (***A***, ***B***) and areas of activation (***C***, ***D***) in areas 3b and area 1 in normal and lesioned conditions. ***A***, ***B***, The total number of digits for amplitude measure is *n* = 25 in normal area 3b (***A***: D1, *n* = 6; D2, *n* = 6; D3, *n* = 6; D4, *n* = 4; D5, *n* = 3), and *n* =19 in normal area 1 (***B***: D1, *n* = 3; D2, *n* = 5; D3, *n* = 3; D4, *n* = 4; D5, *n* = 4), respectively. The total number of digits for amplitude measure is *n* = 7 in lesioned area 3b (***A***: D1, *n* = 2; D2, *n* = 3; D3, *n* = 1; D4, *n* = 1), and *n* = 6 in lesioned area 1 (***B***: D1, *n* = 1; D2, *n* = 2; D3, *n* = 1; D4, *n* = 2), respectively. ***C***, ***D***, The total number of digit pairs (prelesion vs postlesion) for area measure is *n* = 6 in area 3b (***C***: D1, *n* = 2; D2, *n* = 3; D3, *n* = 1), and *n* = 3 in area 1 (***D***: D1, *n* = 1; D2, *n* = 1; D4, *n* = 1). One-way ANOVA test was performed to examine the amplitude differences across digits in normal area 3b (***A***) and area 1 (***B***). ^N.S.^*p* > 0.05, *p < 0.05. Error bars indicate the SE. An unpaired *t* test was performed to compare the amplitude differences between total normal and lesioned digits (total columns in ***A*** and ***B***). Paired *t* test was performed to examine the activation area difference between prelesion and postlesion conditions (total columns in ***C*** and ***D***). *p < 0.05. Error bars indicate the SE.

**Figure 6.**
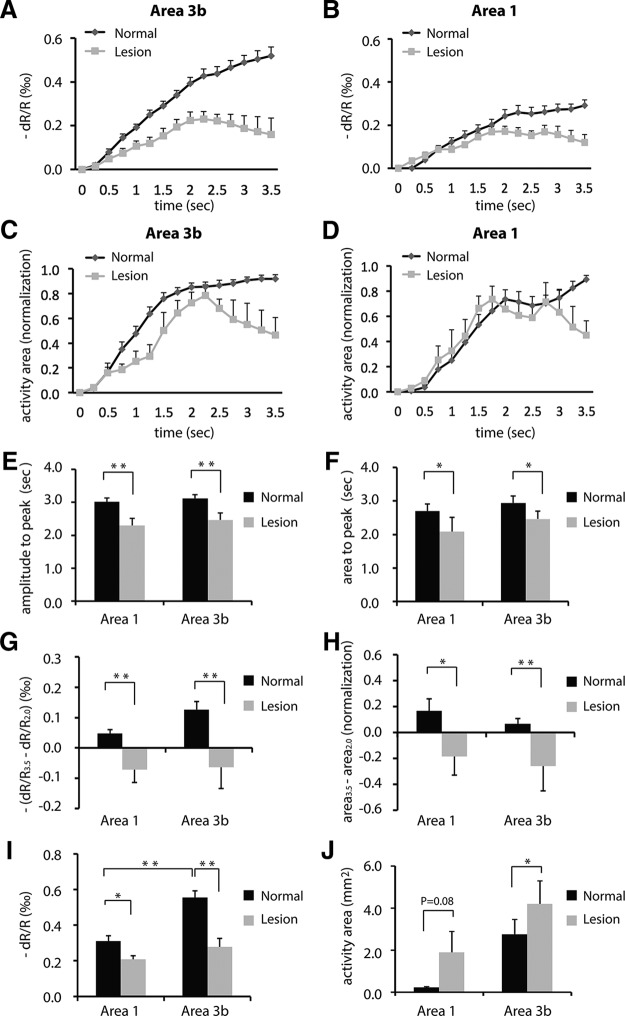
Group-level statistical analysis of the spatiotemporal dynamic features of optical imaging signal changes to single-digit stimulation in normal and lesioned conditions (by DCL) in area 3b and area 1. ***A***, ***B***, Mean time courses of OI signal amplitudes in response to the stimulation of individual digits across all the animals in normal and deafferented conditions in area 3b (***A***) and area 1 (***B***). ***C***, ***D***, Mean time course of activation size changes. ***E***, Time to peak amplitude. ***F***, Time to peak activation size. ***G***, Amplitude changes between 2.0 and 3.5 s after stimulus onset. ***H***, Area changes between 2.0 and 3.5 s after stimulus onset. ***I***, Comparison of peak activation amplitudes in area 1 and area 3b in normal vs lesion conditions. ***J***, Comparison of peak activation sizes in area 3b. Unpaired *t* test for ***E–I*** (normal area 3b: *n* = 25 digits, including 8 digits from prelesion control, 14 digits from normal control, and 3 digits from ipsilateral control. Lesioned area 3b: *n* = 7 digits. Normal area 1: *n* = 19 digits, including 6 digits from prelesion control, 12 digits from normal control, and 1 digit from ipsilateral control. Lesioned area 1: *n* = 6 digits). Paired *t* test was used in ***J*** (area 3b: *n* = 6 digit pairs before and after DCL; area 1: *n* = 3 digit pairs before and after DCL). Details provided in Table 1. *p < 0.05, **p < 0.005. Error bars indicate the SE.

Individual digit and group-averaged OI signal amplitudes, and areas of activation were first calculated to evaluate the variations across digits in normal and lesioned conditions. One-way ANOVA test showed that the OI amplitudes of individual digits (D1–D5) did not differ across digits in area 3b (*p* = 0.71; Fig. 5*A*) and area 1 (*p* = 0.57; Fig. 5*B*) in the normal condition (black columns). However, the variations of the areas of activation in area 3b and area 1 across digits were large. For example, the activity area of D3 in area 3b was about two times the area of other digits in the normal conditions, and was about two times that of D1 and 1.5 times that of D2 in the lesioned condition (Fig. 5*C*). For area 1, the area of D2 activation was approximately one-sixth of the area of D1 and approximately one-fourth that of D4 in the lesioned condition (Fig. 5*D*). Based on these observations, we averaged OI amplitude measures from all digits (D1–D5) at the group level, and compared the differences between normal and lesioned conditions (Fig. 6). For activation area measures, however, only the digits showing activation in prelesion and postlesion conditions (within-digit) were included in the group comparison. The sample size for each measure is provided in the figure legend.

At the group level, we found that the spatiotemporal features of the responses to stimuli in area 3b and area 1 altered significantly after DCL (Fig. 6). The averaged time course of optical signals showed that response amplitude and activation size peaked earlier in both area 3b (Fig. 6*A*,*C*) and area 1 (Fig. 6*B*,*D*) in DCL than under normal conditions. The time to peak amplitude shortened ∼20% (from 3.0 s in the normal condition to 2.3 s in the lesion condition in area 3b, *p* = 0.003; from 3.1 s in the normal condition to 2.5 s in the lesion condition in area 1, *p* = 0.002; Fig. 6*E*). Peak signal amplitude also dropped ∼50% (from 0.55‰ to 0.28‰, *p* = 0.002) for area 3b and ∼30% (from 0.31‰ to 0.21‰, *p* = 0.016) for area 1 (Fig. 6*I*). The signal drop, however, was greater in area 3b than in area 1 after DCL (Fig. 6*I*, compare black–gray pair columns). The signal amplitude differences between normal area 1 and normal area 3b were also significant (*p* = 0.00003), but there was no difference between deafferented area 1 and area 3b (*p* = 0.11, Fig. 6*I*). Spatially, activation size peaked at ∼15% early in area 3b (from 2.9 s in the normal condition to 2.5 s in the lesion condition, *p* = 0.049) and ∼22% early in area 1 (from 2.7 s in the normal condition to 2.1 s in the lesion condition, *p* = 0.04; Fig. 6*F*), as indicated by the time to peak area measures. The activation area was enlarged in both area 1 (*p* = 0.08) and area 3b (*p* = 0.03) after DCL (Fig. 6*J*), but the significant difference was observed only in area 3b. In the later phase of optical signal development, from 2.0 s after stimulus onset to the end of stimulus presentation (3.5 s), both the amplitude and the area of normal area 3b and area 1 continued to increase after peaking, but decreased in deafferented cortices (Fig. 6*G*,*H*). The differences between normal and lesion conditions were statistically significant (amplitude: area 3b, *p* = 0.001; area 1, *p* = 0.0005; area: area 3b, *p* = 0.005; area 1, *p* = 0.029).

### Activation alterations in both anterior–posterior and medial–lateral directions

**Figure 7.**
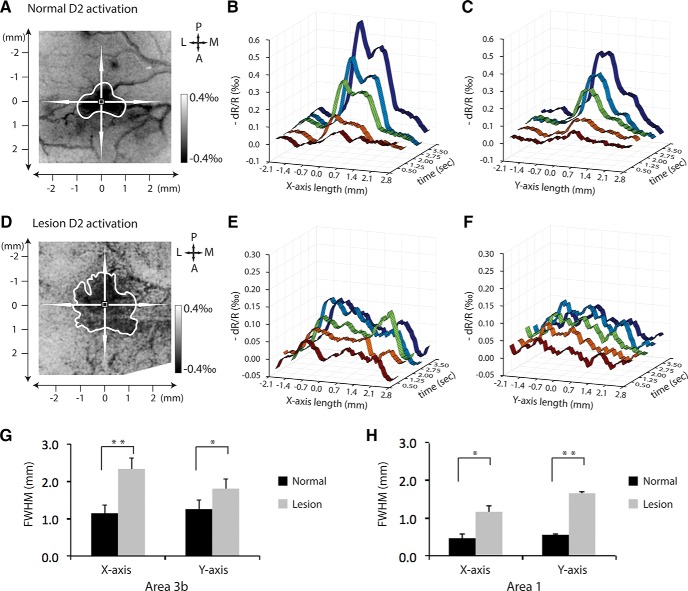
Spatiotemporal dynamics of activation in lateral-to-medial and anterior-to-posterior directions in area 3b in normal vs lesioned conditions. ***A***, D2 activation map in normal cortex in monkey SM-CHA. ***B***, 3D plot of the percentage of optical signal changes as a function of time after stimulus onset (0.5, 1.25, 2, 2.75, and 3.5 s) along the lateral-to-medial axis (*x*-axis) of the activation focus. ***C***, 3D plot of the percentage of optical signal changes as a function of time along the anterior-to-posterior axis (*y*-axis) of the activation focus. ***D***, D2 activation map after lesion. ***E***, 3D plot of the percentage of optical signal changes as a function of time along the lateral-to-medial axis of the activation focus after lesion. ***F***, 3D plot of the percentage of optical signal changes as a function of time along the anterior-to-posterior axis of the activation focus after lesion. ***G***, ***H***, Comparison of the FWHM measures along the two axes of area 3b (***G***) and area 1 (***H***) in normal and lesion conditions. Paired *t* test for ***G*** and ***H***, area 3b pairs = 6 and area 1 pairs = 3. *p < 0.05, **p < 0.005. Error bars indicate the SE.

Because functional organization differs along the medial-to-lateral and anterior-to-posterior directions, we further quantified the FWHM of single-digit activation foci along both the horizontal (medial-to-lateral) and vertical (anterior-to-posterior) axes, and compared the horizontal FWHM and vertical FWHM in prelesion versus postlesion conditions (Fig. 7). 3D plots of signal amplitude as a functional of time (*z*-axis) and space in the horizontal *x*-axis and vertical *y*-axis of the activation foci showed clear extensions of response in both directions after lesion (compare Fig. 7*E*,*F*, 7*B*,*C*). For example, in the normal cortex, optical signals exhibited sharp changes in activation when pixels moved from nonresponsive to responsive regions along the *x*-axis of the plots (Fig. 7*B*,*C*). After lesion, responses lost the sharp border and became wider in space over the entire stimulus presentation period (Fig. 7*E*,*F*). The group results showed that the horizontal FWHM and vertical FWHM were increased significantly in both area 3b (Fig. 7*G*, *p* = 0.001 for horizontal FWHM, and *p* = 0.016 for vertical FWHM ) and area 1 (Fig. 7*H*, *p* = 0.0246 for horizontal FWHM, and *p* = 0.0007 for vertical FEHM) in the lesioned condition. Together, for the summary of the imaging findings described above, see Figure 10*A*.

### Histological evaluation of the extent and spinal cord levels of the unilateral DCL

**Figure 8.**
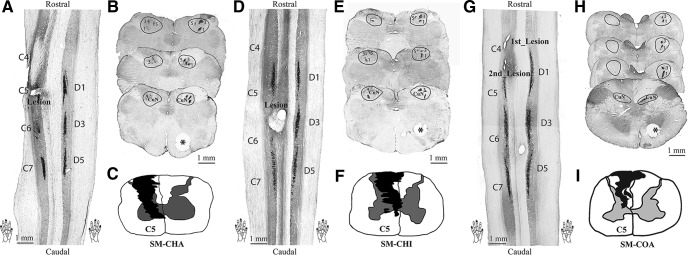
Histological evaluation of CTB-labeled terminations in the spinal cord and cuneate nucleus of the brainstem in three monkeys (***A–C***, SM-CHA; ***D–F***, SM-CHI; and ***G–I***, SM-COA). ***A***, ***D***, ***G***, One horizontally cut CTB-immunoreacted section of the spinal cord showing the location of the lesion and labeled terminal fields after tracer injections. Cervical segments 4**–**7 and foci of label from injections in digits 1, 3, and 5 are marked. ***B***, ***E***, ***H***, Three/four examples of coronally cut CTB-immunoreacted sections through dorsal column nuclei of brainstem. The cuneate nucleus (CuN) is outlined, and numbers 1, 3, and 5 mark the foci of afferents labeled by injections in digits 1, 3, and 5, respectively. Note that there are a few detectable foci of axon fibers on the lesioned (left) side (normal sides are marked with *). ***C***, ***F***, ***I***, Transverse view of spinal cord through cervical segment C5***–***C6 (SM-CHA and SM-CHI), and C4***–***C5 (SM-COA) with the reconstructed extent of the lesion in black. The lesions were reconstructed from a series of horizontally cut sections.

Because the extent and level of afferent disruption are two key factors in determining the severity of behavioral deficits after SCI, we used postmortem histology to evaluate the percentage of afferent disruption after DCL, as well as the level and extent of the injury for all three animals (Fig. 8). Using the appearance of CTB terminal patches of three digits on the intact side of the spinal cord as a control (Fig. 8*A*,*D*,*G*, three black strips on the right sides), the levels of lesion (Fig. 8*A*,*D*,*G*, tissue destruction on the left) were determined to be in the middle portion of D1 for SM-CHA and SM-COA (Fig. 8*A*,*G*), and in the caudal portion of D2 for SM-CHI (Fig. 8*D*). CTB-immunoreacted axial sections of the brainstem also showed that the CTB staining on the lesioned side (left side of the sections) was much weaker and less well organized than the opposite intact side. As shown in Figure 8*B* (SM-CHA), Figure 8*E* (SM-CHI), and Figure 8*H* (SM-COA), three patches of labeled axon terminal fields from the three CTB-injected digits can be identified across sections on the intact side (the right side), but there were only a few sparse patches of label apparent on the lesioned side (the left side), indicating that the lesions were extensive. We further quantified the percentage of afferent disruption by measuring the size of the CTB label in the cuneate nuclei (Fig. 8*B*,*E*,*H*). Using the size of the labeled area in the intact side as a 100% reference, we determined that the afferent disruption was 63% for SM-CHA, 68% for SM-CHI, and 98% for SM-COA. By outlining and reconstructing the locations of missing tissue and adjoining scar tissue on coronal spinal cord sections, we determined the spatial extent of the lesion and its relationship with surrounding spinal cord tissue (Fig. 8*C*,*F*,*I*, black shaded areas). As shown in reconstructed transverse views of spinal cord, the lesion successfully disrupted the dorsal column.

### Behavioral impairment on food reaching and retrieval after DCL

**Figure 9.**
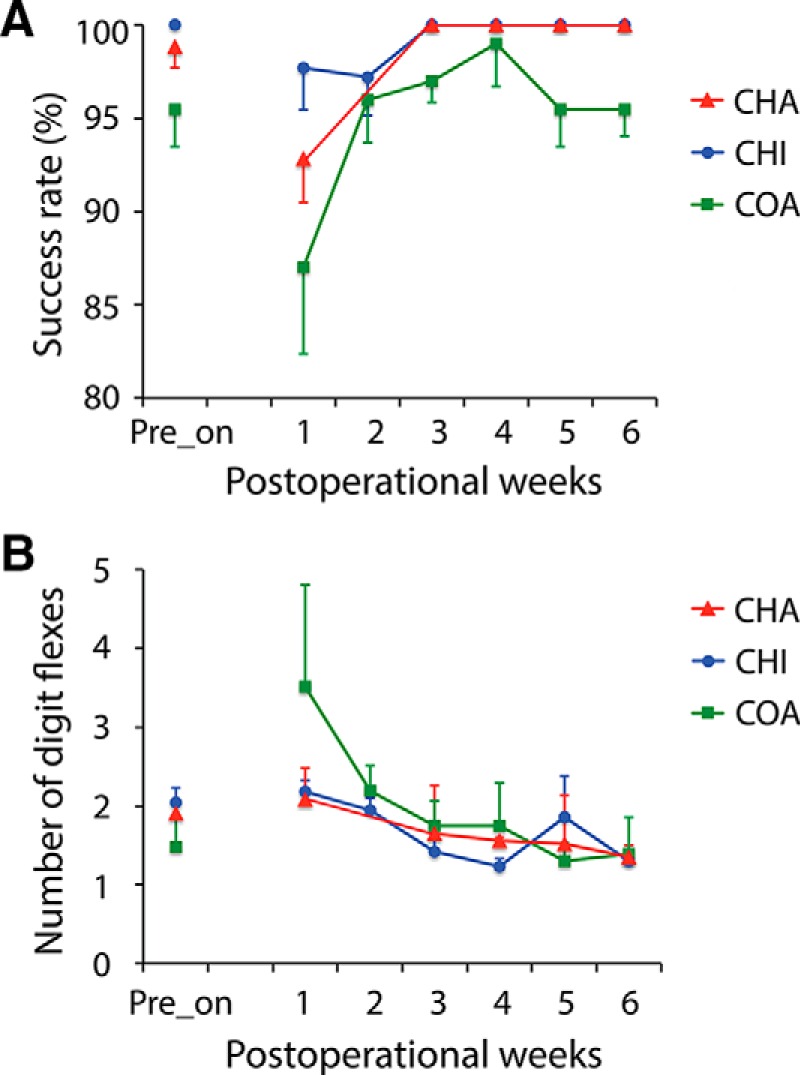
Longitudinal evaluation of food-reaching and food-grasping behaviors following DCL. ***A***, ***B***, Plots of success rates (***A***) and number of flexes (***B***) as a function of different postoperational time points (week) for all three subjects. Error bars indicate the SE.

The effects of DCL on hand use in the home cage and on the food reaching and retrieval task were investigated. For all three monkeys, the affected hands were hardly used for any in-cage activity, such as food reaching and climbing, for the first 3 d immediately after lesion. Within the first 2 postlesion weeks, the hand posture and uses were abnormal. We often observed that animals tended not to use their impaired hand, but effectively did so when forced to use their affected hand to climb cages or grasp and hold food. No other apparent motor deficit was noticed. Behavior performances, as measured by the successful rate and number of flexes of all four well depths, were also quantified before (preoperative) and over a 6 week period after DCL. The most apparent behavioral changes, which are shown in Figure 9, were observed in well 4. For subject SM-CHA, the success rates decreased from 99% prelesion to 93% at the first week and then recovered by week 3 after the lesion (Fig. 9*A*, red dots and lines). The lesion produced a mild increase in the number of digit flexes for the first week in subject SM-CHA (Fig. 9*B*, red dots and lines). In subject SM-CHI, the success rate (Fig. 9*A*, blue dots and lines) and number of digit flexes (Fig. 9*A*, blue dots and lines) dropped slightly for 2 weeks after the lesion, and then recovered by week 3. The largest rate drop was observed in the third subject (SM-COA) at the first week after lesion disrupted 98% afferent. Success rate dropped from 96% prelesion to 87% at the first week, and then recovered by week 3 after SCI (Fig. 9*A*, green dots and lines). The number of flexes increased robustly in the first week after lesion (Fig. 9*B*, green dots and lines). The drop in success rate and the increase in the number of digit flexes returned to prelesion levels at 6 weeks after lesion in all three subjects.

### Schematic summary of the observation

**Figure 10.**
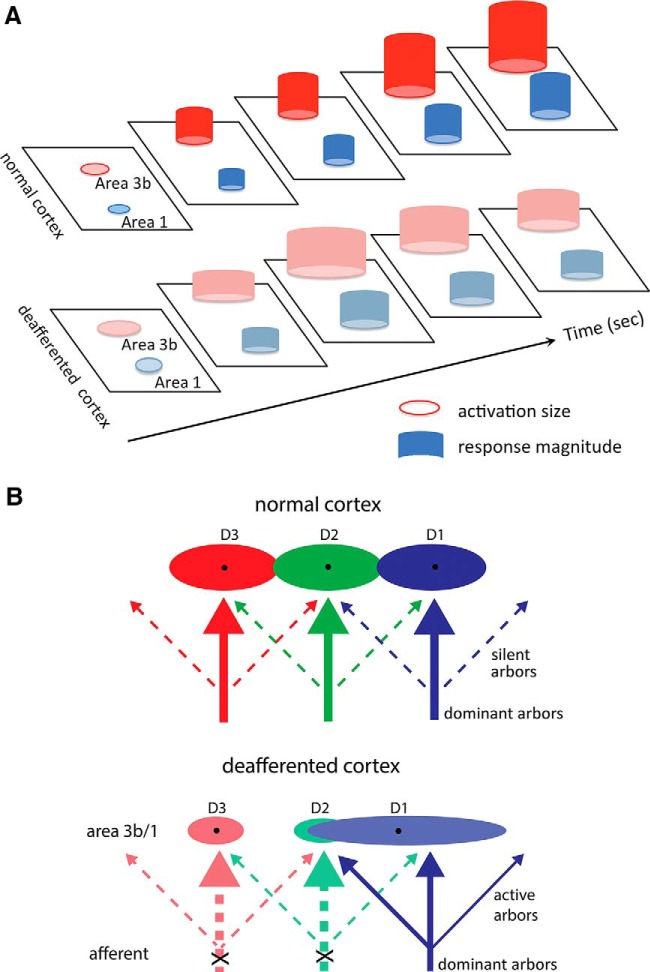
Schematic summary of the spatiotemporal dynamics of optical imaging activations and proposed mechanisms for response property changes after DCL. ***A***, Schematic summary of optical imaging activations to single-digit stimulation in area 3b and area 1 in normal vs deafferented conditions. ***B***, Proposed mechanism for response property changes in reactivated and reorganized digit regions in areas 3b and 1 after partial deafferentation.

The overall observations made in this present study are summarized by the schematic illustration (Fig. 10*A*). Several features are present. In normal input–intact areas 3b and 1 cortex, OI signal amplitude (represented by the height of the cylinder) and the area of activation (represented by the base size of the cylinder) continue to increase during the tactile stimulation period (Fig. 10*A*, top row). After deafferentation, however, the responses of area 3b (Fig. 10*A*, pink cylinders in the bottom row) and area 1 (Fig. 10*A*, light blue cylinders in the bottom row) are weaker than those in the normal condition and more transient, as indicated by the fast OI signal amplitude decline. This finding indicates loss of responses to sustained tactile stimuli. The observation of preferential activation area enlargement for area 3b and area 1 in the medial–lateral direction along digit representation led us to hypothesize that the reduced lateral inhibition of the incoming inputs may be a main contributor to the observed enlarged, but weak, cortical activations in deafferented cortex (Fig. 10*B*).

## Discussion

### Altered information processing of partially deafferented and reactivated area 3b and area 1

Previous studies in the same DCL model showed that reactivation and reorganization of deafferented cortex correlate with the functional and behavioral recovery after SCI (Chen et al., 2012; Qi et al., 2013; Yang et al., 2014). These cortical areas exhibit disorganized and expanded cortical territories and contained neurons with enlarged receptive fields (e.g., multiple digits; Kaas, 2000; Liu et al., 2012). To date, however, little is known about the spatiotemporal dynamics of responses to stimuli in these reactivated areas. A better understanding of these dynamics provides clues about how modules and populations of neurons within and across functionally distinct cortical areas react to deafferentation and work together in space and time to restore or compensate for lost functions. Using the high spatial (100 μm) and temporal (250 ms) resolutions of IOS, we found that areas 3b and 1 responded robustly to tactile stimuli weeks after extensive (63% to 98%) deprivation of ascending afferents, but in significantly altered ways. Responses were enlarged, weakened, peaked early in magnitude and size, and lost sustainability to stimuli. We propose that the characteristic spatiotemporal dynamics in deafferented areas 3b and 1 likely reflect their specific roles in restoring or compensating hand use impairments following DCL.

These new observations, along with the existing knowledge of the functional organization of areas 3b and 1 in tactile representation, led us to propose that the uniformly weakened, diffused, and shortened cortical responses to tactile inputs in partially deafferented areas 3b and 1 likely reflect a combination of compromised ascending inputs, impaired local lateral inhibition, possibly ineffective integration of input activity, and ineffective compensational activity funneling through alternative ascending pathways (e.g., through spared spinothalamic pathway; Qi et al., 2014). Indeed, direct electrophysiology recordings of the reactivated area 3b found that the firing ability of the neurons in response to repeated stimuli were compromised (Wang et al., 2013). Dissociated frequency-dependent stimulus responses of spiking and local field potentials suggest that the integration of local synaptic activity for generating output spiking activity was also compromised. The early peaking of the IOS signals is somewhat more difficult to interpret, given that alternative afferent drives would be expected to take longer to reach the target brain regions of area 3b and area 1. Because optical signals capture more subthreshold synaptic contributions, the early response may reflect enhanced local integrative activities that were absent in normal cortex.

Spatially, the enlarged but diffused responses suggest that more neurons in reactivated areas 3b and 1 were engaged in processing tactile inputs compared with those in normal areas, but the processes were abnormal. IOS signals captured with 630 nm wavelength illumination are sensitive to the deoxyhemoglobin/oxyhemoglobin ratio in superficial cortical layers (i.e., layers 2 and 3; Chen et al., 2003, 2005). Reduced signals may reflect both reduced output spiking and subthreshold synaptic activity. The early occurring IOS signals in border regions of the core digit representation may be indications of unmasked local integrative activity after deafferentation.

Last, differences in spatiotemporal dynamics were apparent between areas 3b and 1 in several aspects. First of all, the greater signal amplitude reduction in area 3b compared with area 1 indicates that deafferentation acted differently in these two areas, despite their close functional relationship in processing tactile inputs. Because area 1 is a higher-order area than area 3b (Vogt and Pandya, 1978; Jain et al., 1997; Romo et al., 2002), the observed differential signal reduction in these two areas suggests that the inputs to area 1 (Cusick et al., 1985) gain synaptic strength and become capable of activating area 1 during recovery from DCL in monkeys. Under such a condition, activation patterns in area 1 become somewhat independent of those in area 3b. One caveat is that the overall activation detection rate in area 1 was lower than that in area 3b (Chen et al., 2005; Friedman et al., 2008). The signal amplitude differences were quantifiable only in the cases where the area 1 activation was detected. Second, the differential response enlargement directions in area 3b versus area 1 suggest that different mechanisms at the local circuit level may be involved in the reactivation and recovery processes. Preferential response engagement of neighboring digit tip regions in area 3b likely reflects the enhanced interactions between digit tips within area 3b in the effort to restore or compensate coordinated digit discriminative ability. In contrast, preferentially engaged digit regions along the digit representation (from tip to base) suggest that area 1 likely plays a key role in restoring the lost tactile sensing ability that requires coordinated activity along the digit tip and phalanxes by enhancing within-digit information integration. Third, the more prominent spatiotemporal alteration of the response in area 3b compared with that in area 1 suggests that the primary input drive may come from subcortical regions (e.g., thalamus), not feedback influences from higher-order brain regions (e.g., S2) after extensive deafferentation. Nevertheless, the present study demonstrates profoundly altered dynamic interactions between deafferented and reactivated digit regions of areas 3b and 1.

Notably, there are some limitations in this study. The first is the limited sample sizes. For example, to maximize the detecting power, we pooled the normal control data from all digits in three different conditions (cortex of normal monkey, prelesioned normal monkey, and ipsilateral cortex of lesioned monkey). Given these condition differences, however, we did not observe significant differences in OI amplitudes in normal conditions, indicating that the interdigit response magnitude difference is small. After deafferentation, group OI amplitude decreases were significant. This finding suggests that deafferentation effects were much more robust than across-digit variation in stimulus responsiveness. In contrast, the area of activation was quite variable across digits. To overcome this issue, we performed only an intradigit (within-digit) comparison to evaluate the effects of deafferentation on the area measured. Under this circumstance, the sample size was quite small, particularly for area 1 where the stimulus response was generally weaker than those in area 3b under anesthesia. We recognize the need for more cases in quantifying activation area changes in spinal cord-lesioned monkeys, particularly for our ultimate goal of linking cortical responses to behavioral impairments and recovery in each individual animal after SCI.

### Behavioral relevance of the reorganization and reactivation of area 3b and area 1 in hand use

For preclinical SCI studies in animals, assessments of clinically relevant and sensitive behavioral measures are crucial for understanding the neuronal mechanisms (e.g., cortical reactivation) mediating the recovery of lost functions and impaired behaviors from injury. In this study, we focused on hand use behavior because it is considered a key factor for improving the quality of life in SCI patients. Hand use in primates involves a higher degree of sensorimotor integration and supraspinal influences than in nonprimate animals (e.g., rats). Accomplishments of complex behaviors such as hand use often involve multiple functionally distinct and hierarchical brain regions (Cools, 1985; Stepniewska et al., 1993; Kambi et al., 2011). Conversely, the recovery of impaired functions or behaviors likely requires collective effort from multiple regions (Wise et al., 1997; Qi et al., 2013). We used qualitative and quantitative behavioral assessments in this study; all three monkeys showed apparent hand use impairments in their in-cage activity during the first 3–4 weeks after SCI. The two measures of success rate and number of flexes quantified in this study were quite variable, and statistically significant differences were detected only within the first week in a subset of subjects. We think that our poor behavioral quantification may be the result of inadequate prelesion baseline behavioral training. We suspect that continued learning confounded the postlesion data. Nevertheless, cortical responsiveness changes, as quantified in this and previous studies, were abnormal for up to months postlesion, when hand use behavior appeared to be normal. The functional and behavioral relevance of these cortical changes in plasticity remains to be determined.

Early recovery of hand use may be the result of a successful compensative strategy that a monkey learns to use when lacking a complete return of somatic sensations. For example, animals would complete the entire reaching-grasping-retrieving behavior without a sugar pellet in hand in some of the testing trials. When it happened, animals would check their hand by eye, and then start another trial. We interpret these false trials (i.e., trial without a target in hand) as compensative behavior when touch sensation is absent or abnormal. We think that in addition to these two measures (i.e., success rate and number of flexes), additional indices are needed for quantifying hand use impairments in conditions that are readily translatable to human studies. Finally, we recognize that our attempts in linking cortical responsiveness changes under anesthesia to awake behavior are limited, and the effects of anesthesia need to be considered (Grandjean et al., 2014; Jonckers et al., 2014). We do not think, however, that the observed dynamic response differences observed before and after SCI were driven by anesthesia variations. Studies have shown that anesthesia has a significantly weaker influence in early sensory cortical areas (for review, see Bonhomme et al., 2011). Nevertheless, direct comparisons of data obtained in the same awake state are needed to solve this potential confounding issue.

### Diffuse cortical response: indication of impaired lateral inhibition in reactivated area 3b and area 1

Lateral inhibition sharpens neuronal response tuning properties, which is essential for fine discriminative ability in sensory systems (von Bekesy, 1967; Merrill and Wall, 1978). Intrinsic cortico–cortical connections in superficial cortical layers are one of the anatomical substrates for lateral inhibition. In normal area 3b, a digit representation region responds to tactile inputs from its corresponding digit, thereby permitting digit-specific information processing. The digit-specific and typically focal activations result from a balanced interaction between a strong drive from the core digit and lateral inhibition from surrounding areas (Brenowitz and Pubols, 1981). In reactivated area 3b and area 1, the more defused and enlarged activation in response to tactile stimuli could indicate compromised lateral inhibition. Two features of the enlarged activation, including the expansion into neighboring regions on a finger (anterior-to-posterior expansion) and across digits (medial-to-lateral expansion), support this interpretation. First, in reactivated area 3b and area 1, digit regions responded to digit stimulation to which they normally would not respond. For example, the D3 region started to respond to inputs from D2 stimulation, but the response was much weaker in magnitude (Fig. 2, compare *Ab*, *Ac*). The weaker and more defused responses may be driven by either previously masked or weak (i.e., subthreshold) input activity. These inputs could come from alternative ascending pathways (e.g., spinothalamic pathway) or interareal feedback. Indeed, the more prominent medial–lateral expansion of activation suggests that lateral inhibition was likely released across the digit tip representations. Extensive digit tip-to-tip intrinsic anatomical connections were identified in previous studies (Négyessy et al., 2013; Wang et al., 2013; Ashaber et al., 2014). The possible mechanism for response property changes in reactivated and reorganized digit regions in areas 3b and 1 after partial deafferentation was proposed in Figure 10*B*. We speculate that impaired lateral inhibition may relate to compromised discriminability, for which we did not test in our animals.

### Implications for functional imaging studies in deafferented cortex

Functional imaging, such as functional MRI, has been widely used in human and animal studies in both physiological and pathological conditions. Because functional imaging signals are of hemodynamic origin and are indirect measures of underlying neuronal activity, an accurate estimation and modeling of the signals are critical for detecting functional signal changes. In this study, the identification of altered dynamic changes of hemodynamic-based optical signal changes to brief peripheral stimuli (3.5 s in duration) in both spatial and temporal domains has significant implications for functional imaging studies of the brain under deafferentation, such as after SCI, stroke, or white matter diseases. Compared with long-lasting tactile stimulation (e.g., 30 s) often used in mapping studies, the use of brief tactile stimulation mimics more closely the engagement of the somatic system during natural sensorimotor behaviors (e.g., hand use), and permits more realistic evaluation of the stimulus response functions. Our findings of profound alterations in spatiotemporal dynamics of the reactivated area 3b and area 1 cortices underscore the need for fine-tuning of hemodynamic response function models in fMRI studies, particularly for those used in event-related designs in pathological deafferentation conditions.

### Conclusions

IOSs with high temporal and spatial resolution revealed altered spatiotemporal dynamics of deafferented and reactivated somatosensory cortical areas 3b and 1 in processing tactile inputs weeks after partial and unilateral DCL. The weaker, more defuse, and more transient IOS responses indicate that reactivated area 3b and area 1 recovered their responsiveness to peripheral natural stimuli, but the information processes at these regions were abnormal. These abnormal activities may contribute to the recovery of simple hand use, but their precise behavioral relevance needs to be further established.

## Synthesis

Reviewing Editor: Trevor Drew, University of Montreal<br />Decisions are customarily a result of the Reviewing Editor and the peer reviewers coming together and discussing their recommendations until a consensus is reached. When revisions are invited, a fact-based synthesis statement explaining their decision and outlining what is needed to prepare a revision will be listed below. The following reviewer(s) agreed to reveal their identity: C. Elaine Chapman

There are questions concerning some of the statistics used in the paper, especially with respect to Figs. 5 and 6. Ideally, the reviewers would have preferred a comparison within subjects rather than between subjects. However, after discussion a consensus was reached that the additional control data (no lesion) should be used to show that there are no differences between digits. If this can be demonstrated, then the pooling (all digits) is justified. Specifically, the authors need to evaluate the extent to which the intensity and area of OI varies across digits (unlesioned and control monkeys pooled). The limitations of this approach also need to be addressed in the Discussion.

Within the limitations of the dataset, the revisions addressed in part my major concern, most especially by clarifying the source of the data (new Table 1) and adding up data from control monkeys. This is an important addition that strengthens the manuscript but these new data were not well incorporated into the manuscript (e.g. new Fig 10 not even referenced in the revised version) and no statistical analyses were performed to strengthen the case that the authors are making. Overall the authors did a poor and minimalist job of revising the manuscript. There remain a number of points not addressed adequately by the authors.

The authors indicate that data from 3 control monkeys are now included in the group quantification of OIS signal amplitude (Fig. 5). This is helpful but the authors must indicate on Fig. 5 the number of observations contributing to each group summary (area 3b, normal, lesioned; area 1, normal, lesioned) - both for signal amplitude and area. The legend should explain the sources of these data (control and experimental monkeys). New Table 1 needs to include the control monkeys.

The new figure (Fig 10) is referenced after Fig. 5, the group summary figure, and before Figs 6-9. The new figure addresses variations in signal amplitude and activation area across digits (normal, lesioned) and areas. Logically this should precede the present Fig. 5 which groups data across digits: new Fig 10 should become new Fig. 5; the numbers of the other figures should be adjusted. The text that describes new Fig 10 is extremely brief and non informative. This needs expansion to describe the contents of the figure, including an explanation as to where the control data are included. Statistical analyses should address the question raised in my review: to what extent are there variations across digits in the experimental measures (signal amplitude, area)? Again the n of observations contributing to each measure needs to be defined (on the figure or in the legend). Finally, in the Discussion, the authors need to address the impact of differences in signal amplitudes/areas across digits and animals to the interpretation of the results.

The authors now state that only paired observations (prelesion, postlesion) were used for comparing areas (Fig. 5). This is a sensible approach. From Table 1, we can see that there were only 3 paired digits for area 1, and these were all in one monkey. How did the authors generate SEMs (Fig. 5) with so few observations? How can they state (see their reply and rerevised text, ln136-7) that "group mean measures are not driven by data from one particular subject" ? This is correct for area 3b but not for the area 1 measures. This limitation must be addressed in the Discussion, and acknowledged in the Abstract.

Minor

1. Most of the data figures (Figs 2,3,4,5,6,10) contain an error: the symbol for percent (%) is incorrect.

2. The y-axis in the plots of reflectance changes vs time (see my point 1) reads: "-dR/R".What is the significance of the negative sign preceding dR/R. This needs to be explained in the Methods and the figure legend.

3. ln 132: please define reflectance (R) here.
